# Glutamate Induces the Elongation of Early Dendritic Protrusions via mGluRs in Wild Type Mice, but Not in Fragile X Mice

**DOI:** 10.1371/journal.pone.0032446

**Published:** 2012-02-27

**Authors:** Alberto Cruz-Martín, Michelle Crespo, Carlos Portera-Cailliau

**Affiliations:** 1 Department of Neurology, David Geffen School of Medicine at UCLA, University of California Los Angeles, Los Angeles, California, United States of America; 2 Department of Neurobiology, David Geffen School of Medicine at UCLA, University of California Los Angeles, Los Angeles, California, United States of America; Nathan Kline Institute and New York University School of Medicine, United States of America

## Abstract

Fragile X syndrome (FXS), the most common inherited from of autism and mental impairment, is caused by transcriptional silencing of the Fmr1 gene, resulting in the loss of the RNA-binding protein FMRP. Dendritic spines of cortical pyramidal neurons in affected individuals are abnormally immature and in Fmr1 knockout (KO) mice they are also abnormally unstable. This could result in defects in synaptogenesis, because spine dynamics are critical for synapse formation. We have previously shown that the earliest dendritic protrusions, which are highly dynamic and might serve an exploratory role to reach out for axons, elongate in response to glutamate. Here, we tested the hypothesis that this process is mediated by metabotropic glutamate receptors (mGluRs) and that it is defective in Fmr1 KO mice. Using time-lapse imaging with two-photon microscopy in acute brain slices from early postnatal mice, we find that early dendritic protrusions in layer 2/3 neurons become longer in response to application of glutamate or DHPG, a Group 1 mGluR agonist. Blockade of mGluR5 signaling, which reverses some adult phenotypes of KO mice, prevented the glutamate-mediated elongation of early protrusions. In contrast, dendritic protrusions from KO mice failed to respond to glutamate. Thus, absence of FMRP may impair the ability of cortical pyramidal neurons to respond to glutamate released from nearby pre-synaptic terminals, which may be a critical step to initiate synaptogenesis and stabilize spines.

## Introduction

In excitatory pyramidal neurons, most synapses are formed on tiny dendritic appendages called spines. Time-lapse imaging experiments have shown that early dendritic protrusions in immature neurons are not only motile and pleomorphic, but also very unstable, turning over quickly over time scales of minutes-hours [1], [2], [3], [4]. This dynamic behavior suggests that immature spines serve an exploratory role, presumably to reach out for appropriate presynaptic partners and initiate synaptogenesis [5]. This notion is supported by data showing that synaptogenesis is impaired following manipulations that alter spine dynamics [6], [7]. We and others have previously demonstrated that focal application of glutamate onto dendrites either recruits new protrusions or causes existing ones to elongate [2], [8], [9]. This suggests that axons might release this excitatory neurotransmitter to attract immature protrusions from nearby dendrites for the purposes of synapse formation. The exact glutamate receptor that mediates these phenomena is not known, though recent evidence suggests NMDA-receptors play a role in glutamate-mediated de novo spinogenesis [9].

However, given that glutamate-mediated elongation of dendritic filopodia is slow (over minutes), it is conceivable that this process might instead be mediated by Group I (GpI) metabotropic glutamate receptors (mGluRs), especially considering that ionotropic receptor blockers do not change protrusion length [2]. Indeed, other studies have shown that application of mGluR agonists causes an elongation of mature dendritic spines in vitro [10], [11].

Whether mGluRs mediate glutamate-induced elongation of dendritic protrusions is an important question in the context of the pathogenesis of fragile X syndrome (FXS), a neurodevelopmental disorder characterized by spine dysgenesis and dysregulated mGluR signaling [12]. We recently showed that immature layer (L) 2/3 cortical pyramidal neurons of Fmr1 knockout (KO) mice [13], a mouse model of FXS, exhibit a delay in the stabilization of dendritic spines during early postnatal cortical development [14]. A different study also reported that dendritic spines of L5 cortical neurons in adult Fmr1 KO mice also display an abnormally high turnover [15]. Symptoms of FXS could therefore partially result from altered synaptogenesis stemming from defects in spine stabilization and perhaps defects in glutamate-mediated elongation of dendritic protrusions.

Here, we used two-photon time-lapse imaging of GFP-expressing neocortical neurons in acute brain slices from neonatal mice to address two questions: First, do mGluRs mediate glutamate-induced elongation of dendritic protrusions? Second, is this phenomenon altered or defective in Fmr1 KO mice? We show that early dendritic protrusions of L2/3 neurons become longer in response to bath application of DHPG, a GpI mGluR agonist. Protrusions also elongate in response to focal puffing of glutamate, but this effect is prevented by blocking GpI mGluR signaling. Importantly, dendritic protrusions from Fmr1 KO mice failed to respond to glutamate.

## Materials and Methods

All experimental protocols (ARC# 2006-016) were conducted according to the National Institutes of Health guidelines for animal research and were approved by the Institutional Animal Care and Use Committee at University California, Los Angeles.

### Mice, constructs and reagents

Fmr1 KO mice in a C57BL/6 background were obtained from Dr. William Greenough (University of Illinois at Urbana Champaign) and bred at UCLA. The experimenters (ACM and MC) were blind to genotyping until after the analysis was completed. Genotypes were determined by PCR analysis of DNA extracted from tail samples using previously described primers [13]. As controls we used either wild type littermates or wild type mice from different litters. A plasmid containing the EGFP coding sequence under the control of the CAG promoter (pCAG-GFP, Addgene plasmid 11150; [16] was used for electroporation. All DNA was purified and concentrated using Qiagen plasmid preparation kits (Valencia, California, United States) and dissolved in 10 mM Tris–HCl.

### *In utero* electroporation

**Figure 1:**
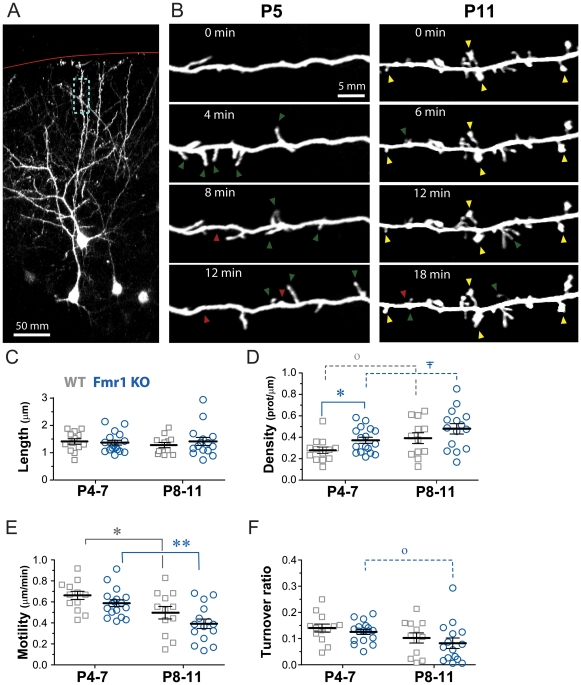
Early dendritic protrusions of Fmr1 KO mice develop normally in vitro, except for a transiently elevated density at P4–7. (A) Low magnification view of L2/3 cells in an acute slice through the somatosensory cortex of a P11 WT mouse (maximum intensity projection of 40 optical slices, 3 µm apart). The cells were sparsely labeled with GFP via in utero electroporation and imaged with two-photon microscopy. The location of the pia is shown by a red line. The boxed region in blue is shown at higher magnification in panel B, right). (B) High magnification view of representative dendritic branches at the two postnatal ages examined. Images are best projections (∼5–11 optical sections, 1 µm apart). Dendrites were imaged every 60 seconds, but only a subset of time points is shown. From P5 to P11, thin protrusions that quickly appear and disappear (green and red arrowheads, respectively) are gradually replaced with more stable spines (yellow arrowheads), typical of mature dendrites, and which often have large swellings (heads) at their tips. (C) Length of dendritic protrusions at two different postnatal ages in WT and Fmr1 KO mice. Each gray square indicates a different dendrite from a WT mouse and each blue circle indicates a different dendrite from a KO mouse. Protrusion length did not change significantly during early postnatal development in WT or KO mice (p = 0.27 and p = 0.79, respectively). (D) Density of dendritic protrusions in WT mice increases by 43% between P4–7 and P8–P11 (^o^ p = 0.05). There was also a nearly significant trend towards higher protrusion density in older KO mice (^∓^ p = 0.06). Compared to WT mice, dendrites in KO mice had a slightly higher density of protrusions at P4–P7 (* p<0.05). (E) Motility of dendritic protrusions decreases between P4–7 and P8–P11 in both WT and KO mice (* p<0.05 and ** p = 0.001, respectively). There were no significant differences in motility between WT and KO mice at either age. (F) Turnover ratio (TOR) of dendritic protrusions does not change significantly in WT mice (p = 0.15), but there was a trend toward lower turnover in older KO mice (^o^ p = 0.05). There were no significant differences in turnover between WT and KO mice at either age.

Progenitor cells of future L2/3 neurons in barrel cortex were transfected via in utero electroporation (Figure 1), as previously described [17]. Timed-pregnant mice at embryonic day (E) 16 were deeply anesthetized using an isoflurane–oxygen mixture (4.5% *[v/v]* for induction and 1.5% *[v/v]* for maintenance) delivered via nose cone using an anesthesia regulator (SurgiVet, Waukesha, Wisconsin, United States). After a midline low abdominal incision the uterine horns were exposed and individual embryos handled gently with forceps. Approximately 1 µl of DNA solution (containing 0.65–1.0 µg/µL of plasmid and 0.1% Fast Green) was pressure injected with a Picospritzer III (Parker Hannifin Corporation, Fairfield, NJ, United States) through the uterine wall into the left lateral ventricle of all embryos using pulled-glass capillaries (Sutter Instrument, Novato, California, United States). Next, the head of each embryo was placed between custom made tweezer-type copper electrodes, and then 2–3 square electric pulses (40 V, 50 ms long) were delivered at 500 ms intervals using a custom built electroporator. The embryos were placed back in the abdominal cavity and the wall of the dam's abdominal cavity and skin were then sutured. After surgery, the dams were allowed to recover in a warm chamber for 1 hour and then returned to their cage.

### Acute slices

Pups at postnatal day (P) 4 through P11 were anesthetized by intraperitoneal injection of ketamine-xylazine and decapitated. The brains were quickly removed and transferred to ice-cold artificial cerebrospinal fluid (ACSF) containing (in mM):126 NaCl, 3 KCl, 3 MgSO_4_, 1.14 NaH_2_PO_4_, 1 CaCl_2_, 26 NaHCO_3_, and 10 dextrose, bubbled with 95% O_2_/5% CO_2_ to a final pH of 7.4. Acute coronal brain slices (300 µm) that included primary somatosensory cortex were obtained using a vibratome (VT1000S; Leica, Bannockburn, IL, United States). The slices were then incubated at 37°C for 15–30 min and a minimum of 30 minutes at room temperature before imaging.

### Pharmacology

**Figure 2:**
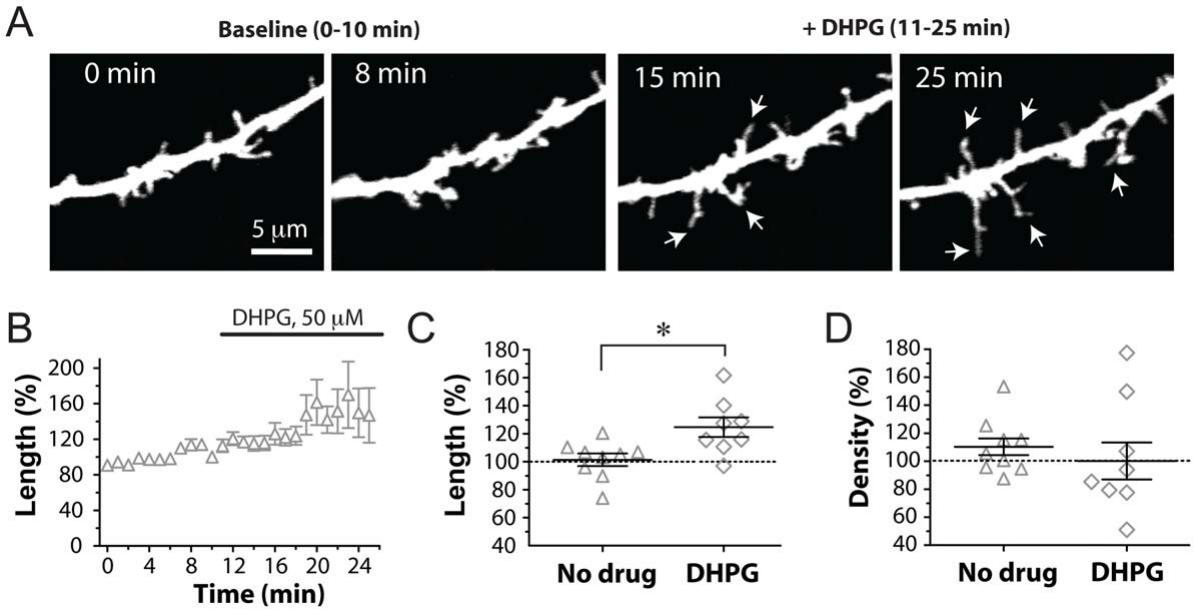
The mGluR5 agonist DHPG elongates early dendritic protrusions in WT mice. (A) Representative images of an apical dendritic segment of a L2/3 neuron from a P6 WT mouse, before (0, 8 min) and after (15, 25 min) bath application of DHPG (50 µM). Images are best projections (∼6–11 optical sections, 1 µm apart). Dendrites were imaged every 60 seconds for 25 min, but only a few representative time points are shown. (B) Change in length of protrusions over time, before and during bath application of DHPG. The data is normalized to the average of the baseline time points (0–10 min). (C) Change in length of protrusions before and after bath application of DHPG or ACSF alone (“No drug”). Data from the last 21–25 min are compared to the baseline 0–5 min for each condition. The mGluR agonist caused a 24% increase in the average length of protrusions, compared to ACSF alone (* p<0.05). (D) DHPG did not change the density of dendritic protrusions (p = 0.47).

**Figure 3:**
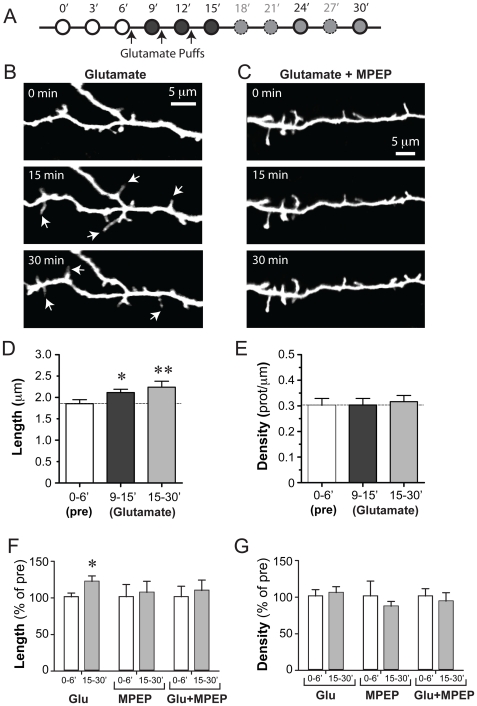
Blockade of mGluR5 prevents glutamate-mediated elongation of dendritic protrusions. (A) Experimental design. Using a Picospritzer we delivered 5 quick puffs of glutamate (200 msec duration, 800 msec interval) onto the dendrites at 3 different times (black arrows; 3 min apart) beginning after a baseline imaging period of 6 min. Images were acquired every 3 min for the duration of the experiment, but we only analyzed data at 0, 3, 6, 9, 12, 15, 24 and 30 min. (B) Representative images showing a dendritic segment of a L2/3 neuron from a P7 WT mouse before (0 min) and after (15 and 30 min) puffing of glutamate (200 µM). Images are best projections (4–7 optical sections, 1 µm apart). Note how several protrusions elongated in response to glutamate (white arrows). (C) Representative images showing a dendritic segment of a L2/3 neuron from a P8 WT mouse before and after puffing of glutamate (200 µM) in the presence of the mGluR5 inverse agonist MPEP (50 µM). Images are best projections (6–9 optical sections, 1 µm apart. The glutamate-mediated lengthening of protrusions was blocked by MPEP. (D) Length of protrusions before and after application of glutamate. Only protrusions that were present for at least 2 time points before puffing and for at least 2 time points after puffing were included in this analysis. Glutamate caused a 18% and 21% increase in the average length of protrusions at 9–15 min and 15–30 min, respectively (* p = 0.05; ** p<0.005). (E) Density of protrusions before and after application of glutamate. All protrusions present during imaging were included in this analysis. There were no significant changes after glutamate (p>0.39). (F) Changes in length of protrusions (as a percentage of the 0–6 min baseline) at 15–30 min after glutamate puffing, bath application of MPEP alone, or puffing glutamate in the presence of MPEP. Only the glutamate-induced 21% increase in the length of protrusions was significant (p<0.05, Dunnet's multiple comparison test). (G) Changes in density of protrusions (as a percentage of the 0–6 min baseline) at 15–30 min for the same three conditions. There were no significant changes (p>0.05, repeated measures ANOVA).

For stimulation of GpI mGluRs we bath applied the selective agonist (*S*)-3,5-dihydroxyphenylglycine (DHPG; Tocris Bioscience, Ellisville, MO, United States) at 50 µM, after a baseline imaging period of 10 min. Controls for these experiments (“no drug” in Figure 2) consisted in imaging under standard ACSF conditions for the entire duration of the experiment. For glutamate puffing experiments, a glass microelectrode filled with 200 µM glutamate (dissolved in ACSF and containing 0.05 mM Alexa-488 to visualize the pipette while imaging) was lowered into the slice and placed within 10–50 µm away from the dendrite of interest. Using a PicoSpritzer, we delivered 5 puffs of glutamate (200 msec duration, 800 msec interval, 15 psi) at 3 different times (3 min apart) beginning after a baseline imaging period of 6 min (see Figure 3A), which is similar to what we did previously [2]. 2-methyl-6-phenylethynyl pyridine hydrochloride (MPEP, 50 µM; gift from the FRAXA Research Foundation) was bath applied starting 20 min before imaging began and throughout the imaging session, including during the glutamate puffing.

### Imaging

All imaging was performed with a custom-built two-photon microscope, using a Ti∶Sapphire laser (Chameleon XR, Coherent Inc., Santa Clara, California, United States) tuned to 910 nm. The objective (40X, 0.8 NA water immersion), tube lens and trinoc were from Olympus (Tokyo, Japan) and the photomultiplier tube from Hamamatsu (Hamamatsu City, Japan). For imaging, we used ScanImage software [18] written in MATLAB (MathWorks, Natick, Massachusetts, United States). Excitation power measured at the back aperture of the objective was typically between 20 to 40 mW and was adjusted to achieve near identical levels of fluorescence within each imaging session. During an imaging session, 1–5 dendritic regions of interest (ROI) per brain slice were selected along apical dendrites of GFP-expressing L2/3 pyramidal neurons. Each dendritic ROI was collected at high ScanImage zoom (7–9x) and consisted of a stack of images (∼5–20 optical sections, each separated axially from the next by 1 µm), where the resolution for each optical section (512×512 pixels) was 0.12–0.15 µm/pixel. For time-lapse imaging of the developmental time course of dendritic protrusions in WT and KO mice and for DHPG experiments, we collected stacks every 60 seconds over 15–30 min. For glutamate puffing experiments we imaged slices every 3 min. Care was taken to achieve close to identical fluorescence levels across imaged regions within an experiment and across different imaging time points. In some of the figure panels, distracting processes (e.g., axons and dendrites from other neurons) were digitally removed in Adobe Photoshop for display purposes only.

### Data analysis

**Figure 4:**
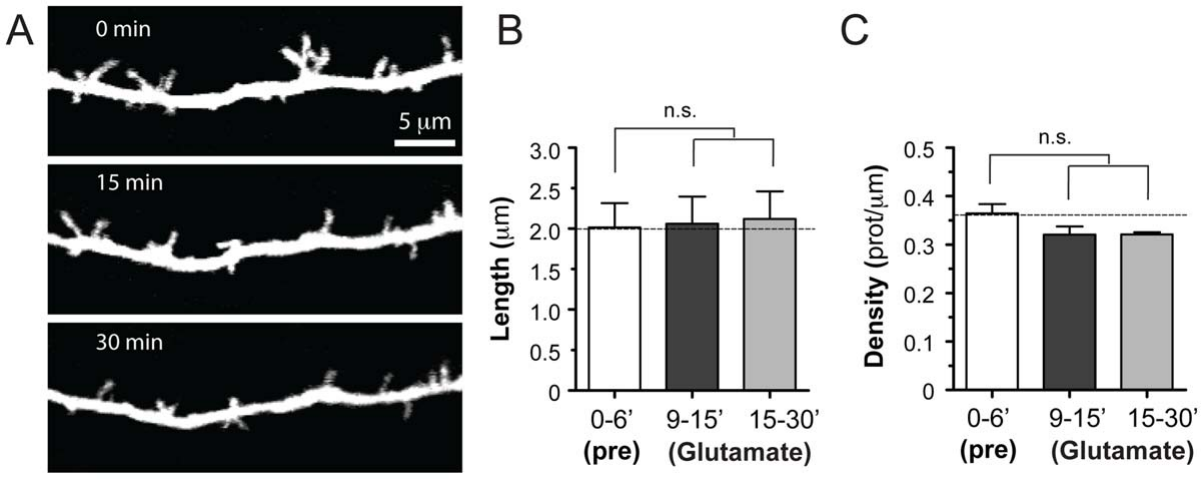
Early dendritic protrusions of Fmr1 KO mice are insensitive to glutamate. (A) High-resolution images of dendritic protrusions from a Fmr1 KO mouse at P9, before (0 min) and after (15 and 30 min) puffing glutamate (200 µM). Images are best projections of 8–11 optical sections, 1 µm apart. (B) Length of protrusions in Fmr1 KO mice before and after puff application of glutamate (analysis was the same as in Figure 3). The average length of protrusions was not changed by glutamate (p = 0.78). (C) Density of protrusions before and after application of glutamate. There were no significant changes after glutamate (p = 0.12).

For the developmental time course experiments in WT mice (Figure 1) we analyzed 13 and 12 dendrites (from 8 mice each) at P4–7 and P8–11, respectively. For the developmental time course experiments in Fmr1 KO mice (Figure 1), we analyzed 17 and 15 dendrites from 8 and 4 mice at P4–7 and P8–11, respectively. For the DHPG experiments (Figure 2), we analyzed 8 dendrites from 6 different mice treated with 50 µM DHPG and 9 control dendrites from 8 mice without DHPG (“no drug” in Figure 2). For glutamate and MPEP experiments in WT mice (Figure 3), we analyzed 2 dendrites from 2 mice exposed with ACSF alone, 9 dendrites from 8 mice exposed to glutamate alone, 3 dendrites from 3 mice exposed to MPEP alone, and 6 dendrites from 4 mice exposed to glutamate+MPEP. For glutamate puffing experiments in KO mice (Figure 4), we analyzed 5 dendrites from 4 mice.

Data on dendritic protrusion length, density and dynamics was obtained using protrusion analysis software written in MATLAB (kindly provided by Tim O'Connor and Karel Svoboda, Janelia Farm, HHMI). This analysis is done on the raw image stacks (except for a median filter, radius = 1) and the presence or absence of a protrusion was determined by inspecting individual slices from the entire stack of images. However, because of the lower resolution of two-photon microscopy in the axial plane, only dendritic protrusions that were clearly projecting laterally were included in the analysis [19]. For a dendritic protrusion to be considered new or lost it had to clearly protrude out of the shaft by at least three pixels (>0.45 µm), which corresponded to the noise on either side of the dendritic shaft. Measurements of protrusion length (from protrusion head to shaft) were accomplished by manually drawing a line through the center of the protrusion to the shaft of the dendrite for each frame the protrusion was present, divided by the total number of frames. Motility was calculated as the absolute difference in length of protrusions from frame-to-frame, divided by the total number of frames. Protrusion turnover (per 60 sec) was defined as the number of protrusions lost+the number of protrusions gained, divided by twice the total number of protrusions.

All statistical analyses were performed with GraphPad Prism (GraphPad Software Inc., La Jolla, California, United States) and error bars in graphs represent the standard error of the mean (s.e.m.). To determine statistical significance, we used either an unpaired two-tailed Student t-test (Figures 1C–F, 2D–E, 3F–G), or a one-way repeated measures ANOVA followed by Dunnett's multiple comparison test (Figures 3D–E and 4B–C). Significance was set at p<0.05.

## Results

### Developmental regulation of early dendritic protrusions *in vitro*

We examined various aspects of the maturation of dendritic protrusions in L2/3 pyramidal neurons from WT and KO mice during early postnatal cortical development with two-photon microscopy in acute brain slices, similarly to our previous study of L5 neurons [2]. L2/3 neurons were labeled with GFP using in utero electroporation in mice at E16. This resulted in a sparse labeling of neurons, which was ideal for high-resolution two-photon imaging of dendritic spines (Figure 1A). In a series of control experiments, we previously characterized the action potential firing and passive membrane properties of GFP-transfected neurons and found them to be indistinguishable from those of untransfected neighboring neurons [14].

For imaging experiments, we focused on the first two postnatal weeks because this time period is characterized by the initial stages of synapse formation [20] and a critical period for experience-dependent fine tuning of intracortical circuitry [21], [22], [23], [24], [25]. This age range also includes the time when we reported an abnormally high rate of protrusion turnover in Fmr1 KO mice [14]. In order to investigate whether similar alterations in protrusion dynamics occur in vitro, we performed time-lapse imaging of dendritic branches of L2/3 neurons at 60 sec intervals.

In acute slices from WT mice, dendritic protrusions followed the expected changes in density, shape and size throughout postnatal development (Figure 1B). At the earliest ages imaged (P4–7) there was an abundance of long and headless protrusions, as previously described for immature neurons in vitro [1], [2], [26]. But by P8–11, many protrusions resembled mushroom-like spines, which are typical of mature dendrites [27]. Quantitative analysis demonstrated a slight decrease in the length of protrusions between P4–7 and P8–11, but it was not statistically significant (1.44±0.08 vs. 1.29±0.09 µm, n = 13 and 12 dendrites at P4–7 and P8–11, respectively; p = 0.27, t-test; Figure 1C). The density of protrusions was 43% higher in the older age group (0.28±0.03 vs. 0.40±0.05 protrusions/µm; p = 0.05, t-test; Figure 1D). Compared to in vivo imaging data for L2/3 neurons at similar ages [14], the density of protrusions in acute slices was similar.

The time-lapse imaging experiments allowed us to quantify dynamic parameters, such as protrusion motility and turnover. Early dendritic protrusions in WT mice were 30% more motile at P4–7 than at P8–11 (0.66±0.04 vs. 0.50±0.06 µm/min, p = 0.03, t-test; Figure 1E), as has been reported previously [2], [26]. In addition to being highly motile, protrusions of immature neurons quickly emerged and disappeared from dendrites over a scale of minutes. The turnover ratio of protrusions was slightly higher at P4–7 than at P8–11, but this difference was not significant (p = 0.15, t-test; Figure 1F). Of note, protrusion turnover for L2/3 neurons is much higher in acute brain slices than in vivo, where protrusions appear and disappear over time scales of tens of minutes [14], which underscores the effects of slicing on protrusion dynamics and synaptogenesis.

### Transiently higher density but normal length of protrusions in Fmr1 KO mice *in vitro*

Next we examined whether the developmental maturation of dendritic spines is altered in acute brain slices from Fmr1 KO mice. Data on spine density and length in Fmr1 KO mice are somewhat controversial, even during early postnatal development [28]. One study that examined L5 cortical neurons in fixed tissue from neonatal Fmr1 KO mice found that the density and length of spines are abnormally high in the first postnatal week, but both are normal by 4 weeks of age [29]. In contrast, when spines were imaged in living neurons in organotypic slices the same authors reported no differences in spine density or length compared to WT mice. Similarly, two recent live imaging studies of cortical pyramidal neurons failed to detect differences in spine density in neonatal, juvenile or adult Fmr1 KO mice [14], [15].

We imaged early dendritic protrusions in acute brain slices of Fmr1 KO animals. Just as with WT mice, the length of protrusions in KO mice remained stable throughout the first two postnatal weeks (1.37±0.09 at P4–7 vs. 1.41±0.15 at P8–11, p = 0.79, t-test; Figure 1C), and there were no significant differences compared to WT at either age (p>0.52, t-test). In addition, KO mice also exhibited a strong developmental trend toward higher protrusion density in older mice (from 0.37±0.03 protrusions/µm at P4–7 to 0.48±0.05 protrusions/µm at P8–11; p = 0.06, t-test; n = 17 and 15 dendrites at P4–7 and P8–11, respectively; Figure 1D). Interestingly, the density of dendritic protrusions was slightly higher in KO than in WT mice but the difference was only statistically significant at P4–7 (0.28±0.03 protrusions/µm in WT vs. 0.37±0.03 protrusions/µm in KO, respectively; p = 0.04, t-test; Figure 1D).

### Normal dynamics of dendritic protrusions in Fmr1 KO mice *in vitro*

We recently showed that L2/3 neurons from Fmr1 KO mice have an abnormally high turnover of early dendritic protrusions in vivo at P10–12 [14]. Here, we examined protrusion dynamics in vitro using time-lapse imaging of L2/3 neurons at 1 min intervals. A previous study failed to detect changes in motility of protrusions in 7-day old L5 neurons imaged in organotypic slices at 2-min intervals [29]. Just as in WT mice, protrusion motility in KO mice decreased over the first 2 postnatal weeks (0.59±0.03 µm/min at P4–7 vs. 0.39±0.05 µm/min at P8–11; p = 0.001, t-test), but there were no differences compared to WT mice at either age (p>0.15, t-test; Figure 1E). We also observed a slight developmental downregulation of protrusion turnover in KO mice (0.13±0.01 per min at P4–7 vs. 0.08±0.02 at P8–11, p = 0.05, t-test; Figure 1F), but there was no difference between KO and WT mice at either age (p>0.43, t-test).

### Glutamate-induced elongation of dendritic protrusions is mediated by group I mGluRs

Previous work has demonstrated that the mGluR agonist DHPG can elongate mature dendritic spines of dissociated hippocampal neurons in culture and/or granule cells in organotypic hippocampal slice cultures (∼3 weeks in culture) [10], [11]. To test whether this is also the case for less mature dendritic protrusions of neocortical pyramidal neurons in a more intact neuronal circuit, we examined the effects of DHPG on apical dendrites of L2/3 neurons in acute slices (age range P4–P10, average 6.6 days of age). Bath application of DHPG (50 µM) resulted in a 24% increase in the average length of dendritic protrusions compared to slices incubated in ACSF alone (Figure 2; p = 0.01, t-test, n = 8 and 9 dendrites, respectively), but had no effect on protrusion density (p = 0.48).

Next, we investigated whether the glutamate-induced elongation of dendritic protrusions that we previously observed on L5 pyramidal neurons (Portera-Cailliau, 2003) is a generalized phenomenon that also affects L2/3 neurons, and whether it could be prevented by blocking GpI mGluRs. We pressure injected glutamate (200 µM) onto apical dendritic segments of L2/3 neurons (age range P7–P9, average 7.9 days of age) during high-resolution time-lapse imaging sessions, at 3 min intervals after a baseline 6 min-long period of imaging (see Methods; Figure 3A). When we analyzed all protrusions that were present throughout the entire imaging period (0–30 min), including new ones that appeared during imaging and those that disappeared before glutamate puffing, we observed an increase in the length of all protrusions, but it did not quite reach statistical significance (1.60±0.09 µm at 0–6 min vs. 1.66±0.08 µm at 9–15 min and 1.80±0.10 µm at 15–30 min; p = 0.076, repeated measures ANOVA; n = 9 dendrites, 172 protrusions; not shown). However, when we analyzed only those protrusions that were present for at least 2 baseline time points immediately before puffing (i.e., those that were exposed to puffing) and persisted for at least 2 time points after puffing, we found that glutamate led to a significant increase in the length of protrusions both immediately after application (2.11±0.08 µm at 9–15 min vs. 1.85±0.10 µm at 0–6 min; 18% increase; n = 9 dendrites, 59 protrusions; p<0.05, Dunnett's multiple comparison test) and up to 30 min after puffing (2.22±0.14 µm at 15–30 min; 21% increase; n = 9 dendrites, 53 protrusions; p<0.005, Dunnett's multiple comparison test; Figure 3B and D). In contrast, puffing ACSF had no effect on protrusion length (not shown; n = 2 dendrites, 27 protrusions, from 2 mice), suggesting that the glutamate effect was not purely mechanical (e.g., due to displacement of the dendrite). Glutamate puffing did not affect the density of protrusions (0.32±0.02 protrusions/µm at 15–30 min vs. 0.30±0.03/µm at 0–6 min; p>0.05, repeated measures ANOVA; n = 9 dendrites, 172 protrusions; Figure 3E).

We then tested whether this glutamate-induced elongation of dendritic protrusions could be prevented by bath application of MPEP (50 µM), which acts as a potent non-competitive mGluR5 antagonist by inhibiting both agonist-induced activation of the receptor and its constitutive activity [30]. For these experiments with MPEP we again analyzed only those protrusions that were present for at least 2 baseline time points immediately before puffing. MPEP alone (age range P6–P9, average 8 days of age) had no effect on the length or density of dendritic protrusions (p>0.05, repeated measures ANOVA, 0–6 min vs. 9–15 min vs. 15–30 min; n = 3 dendrites, 62 protrusions exposed to MPEP throughout the imaging period). When brain slices were pre-incubated in the presence of MPEP for 10–20 min before glutamate puffing (age range P6–P10, average 7.8 days of age), glutamate failed to elongate dendritic protrusions (Figure 3C, F; p>0.05, repeated measures ANOVA, 0–6 min vs. 9–15 min vs. 15–30 min; n = 6 dendrites, 29 protrusions that were present throughout the baseline and glutamate puffing). When we compared the effects of glutamate alone, MPEP alone, or glutamate in the presence of MPEP to one another, only the effects of glutamate on baseline protrusion length were significant (Figure 3F; p<0.05, Dunnett's multiple comparison test, baseline vs. 15–30 min).

### Dendrites of L2/3 neurons in Fmr1 KO mice are insensitive to glutamate

It has been proposed that unchecked signaling though mGluRs in Fmr1 KO mice could account for many of the neurological and psychiatric aspects of FXS [31]. This might conceivably result in exaggerated glutamate-induced lengthening of dendritic protrusions, so we tested how dendritic protrusions of L2/3 neurons in Fmr1 KO mice respond to glutamate (age range P7–P9, average 8.2 days of age). Again, we analyzed only those protrusions that were present for at least 2 baseline time points immediately before puffing, just as with WT mice in Figure 3. Unexpectedly, we found that dendritic protrusions of Fmr1 KO mice were insensitive to focal puff application of glutamate, as their length did not change up to 30 min after puffing (2.06±0.33 µm at 9–15 min and 2.12±0.34 µm at 15–30 min, vs. 2.01±0.03 µm at 0–6 min; p>0.05, repeated measures ANOVA; n = 5 dendrites, 34 protrusions that were present throughout the baseline and glutamate puffing; Figure 4B). Although average protrusion length was slightly higher in the mutant mice (compare baselines in Figure 3D with Figure 4B), the difference was negligible and not statistically significant. The overall density of protrusions in KO mice also remained unchanged after glutamate (0.32±0.01 protrusions/µm at 15–30 min vs. 0.36±0.02 protrusions/µm at 0–6 min; p>0.05, repeated measures ANOVA; n = 5 dendrites, 88 protrusions; Figure 4C).

## Discussion

### Regulation of early dendritic protrusions by glutamate

One of the main findings in this study is that immature dendritic protrusions of L2/3 cortical pyramidal neurons elongate in response to focal glutamate application. Previously, it had been shown that glutamate can elongate dendritic protrusions in cultured hippocampal neurons [8], [32], and we also demonstrated that the same holds true for L5 pyramidal neurons in acute slices [2]. We conclude that this is a widespread phenomenon for excitatory neurons during the development of cortical structures. It seems intuitive that glutamate released by axons might recruit immature protrusions of nearby dendrites to initiate synaptogenesis. This idea is further supported by recent work showing that glutamate uncaging onto dendritic shafts of immature L2/3 neurons in slices can lead to de novo spinogenesis [9], a finding that echoed results obtained through electrical stimulation in hippocampal neurons [33]. The change in protrusion length triggered by glutamate that we previously reported for L5 neurons was more dramatic (75% longer; [2]) than what we now see for L2/3 neurons (21% longer). We suspect the difference is because at the older ages that we imaged L2/3 neurons the neuropil is more densely packed, which may impose a physical constraint for spine growth. A crucial question that remains to be addressed is whether glutamate also induces spine growth/elongation in vivo.

One of the goals of the present study was to identify the signaling pathway that mediates this glutamate-induced elongation of dendritic protrusions. Because the process evolves slowly over minutes rather than seconds, we hypothesized that mGluRs might be involved. In addition, others had already found that the group I mGluR agonist DHPG causes an elongation of mature dendritic spines in neurons in culture [10], [11]. Here we show that DHPG causes a similar elongation of immature dendritic protrusions without affecting their density (Figure 2). Furthermore, we show that glutamate-mediated elongation of dendritic protrusions is blocked by MPEP, a specific mGluR5 blocker. Our results contrast with those of Kwon et al. (2011), demonstrating that MPEP and other blockers of GpI mGluR signaling did not prevent glutamate-mediated spinogenesis, while an NMDA-R antagonist did. It is certainly possible that the generation and elongation of spines are two different phenomena that are regulated by different pathways. Interestingly, we previously found that chronic bath application of APV (and CNQX) to neonatal cortical slices led to a decrease in spine density in L5 neurons [2], which fits with the Kwon et al. data (2011). It is also worth noting that our glutamate experiments were done in slightly younger mice and also that differences in how glutamate is applied (uncaging focally vs. puffing over a large dendritic segment) could have different effects on protrusions.

### Normal developmental regulation of dendritic protrusion dynamics in Fmr1 KO in slices

Two groups recently demonstrated, independently, that dendritic spines of cortical pyramidal neurons are abnormally unstable in developing and adult Fmr1 KO mice, compared to WT mice [14], [15]. Considering the evidence supporting a an important role for FMRP in experience-dependent synaptic plasticity [15], [34], [35], [36], we wondered whether similar defects in turnover are also apparent in vitro. However, our time-lapse imaging of L2/3 neurons in acute brain slices failed to detect any differences in the length or dynamics of dendritic protrusions between WT and KO mice during early postnatal development. The only difference we identified was a transiently elevated spine density in KO mice at P4–P7. This is in contrast with two studies that reported a normal spine density for L2/3 and L4 pyramidal neurons in fixed slices at early postnatal ages [35], [37]. In addition, in our recent in vivo imaging study, we reported that spine density was normal in KO mice throughout postnatal development [14]. However, our slice data are in agreement with a prior study in fixed tissue that reported a transiently higher density of dendritic spines in L5 pyramidal neurons from barrel cortex of KO mice during the first postnatal weeks [29]. Curiously, that same study failed to detect differences in protrusion density or length using live imaging in cultured slices of the same age [29]. These conflicting results underscore the impact of methodological differences on results of spine density in KO mice and also the differences between studies done in vivo and in vitro. The latter is particularly striking for spine turnover, which can be an order of magnitude higher in vitro than in vivo (compare our data presented here and that in the Cruz-Martin 2010 study). Indeed, spine dynamics are regulated by sensory experience [22], [38], so removing peripheral inputs during brain slicing likely explains these differences. Another possible explanation is that the slicing procedure itself could lead to changes in spine turnover, as it also affects their density [39], [40]. Importantly, our data at P10–12 are in agreement with several other studies that have found normal spine density for pyramidal neurons in L2/3, L4 or L5, at 2 weeks of age, especially studies using imaging in living neurons [14], [29], [37].

### Protrusions in Fmr1 KO mice do not respond to glutamate: implications for synaptogenesis

The mGluR theory of FXS has proposed that many of the anatomical, circuit plasticity and cognitive-behavioral defects of Fmr1 KO mice could be explained by altered (excessive) signaling through mGluRs [31]. This is certainly true in hippocampus, and recent evidence suggests that enhanced mGluR5 signaling may also occur in the neocortex of mutant mice [41]. This prompted us to examine whether mGluR-mediated elongation of early dendritic protrusions might be deficient in Fmr1 KO mice. We found that early dendritic protrusions of L2/3 neurons from KO mice, unlike those of WT mice, did not elongate in response to glutamate. Intuitively, one might have expected that dendrites from Fmr1 KO mice would have an exaggerated response to glutamate application and therefore that protrusions in KO mice would have been even longer after glutamate puffing. One possible explanation is that the mGluR signaling pathway is saturated in Fmr1 KO mice and protrusions cannot elongate further with glutamate puffing. But this is unlikely considering that protrusion length was normal in the mutant mice at both ages (Figure 1C). Another possibility is that the defects in mGluR signaling may be different in the neocortex than in the hippocampus of Fmr1 KO mice. Although our data do support the notion that mGluR signaling may be disrupted in the barrel cortex of mutant mice, additional studies will be needed to determine whether mGluR signaling is also upregulated in neocortex, and whether the alteration is a primary defect from loss of FMRP or a compensatory phenomenon.

We hypothesize that glutamate-induced elongation of early protrusions is probably a crucial step to initiate synaptogenesis. The fact that L2/3 neurons in Fmr1 KO mice are insensitive to glutamate might explain the apparent reductions in spine synapses that have been reported in the mutant mice [42], [43]. In addition, it might explain why spine turnover is abnormally high in Fmr1 KO mice, because even the slightest problems in forming early synapses could result in a failure to stabilize spines during the peak period of synaptogenesis in early postnatal development [14]. Given that perturbations in protrusion dynamics result in altered synaptogenesis [6], [7], this may result in a vicious cycle of altered synaptogenesis in FXS. Future studies will have to address how FMRP, which is an RNA-binding protein that controls the translation of several mRNAs that regulate spine shape, receptor trafficking, synaptogenesis, and synaptic plasticity [44], [45], [46], influences the maturation and dynamics of early dendritic protrusions.
